# XTEND: Extending the depth of field in cryo soft X-ray tomography

**DOI:** 10.1038/srep45808

**Published:** 2017-04-04

**Authors:** Joaquín Otón, Eva Pereiro, José J. Conesa, Francisco J. Chichón, Daniel Luque, Javier M. Rodríguez, Ana J. Pérez-Berná, Carlos Oscar S. Sorzano, Joanna Klukowska, Gabor T. Herman, Javier Vargas, Roberto Marabini, José L. Carrascosa, José M. Carazo

**Affiliations:** 1 Centro Nacional de Biotecnología (CNB-CSIC), Cantoblanco, Madrid, 28049, Spain; 2 ALBA Synchrotron Light Source, Cerdanyola del Vallès, Barcelona, 08290, Spain; 3 Centro Nacional de Microbiología, ISCIII, Majadahonda, Madrid, 28220, Spain; 4 Department of Computer Science, The Graduate Center, City University of New York, NY 10016, USA; 5 Escuela Politécnica Superior, Univ. Autónoma de Madrid, Cantoblanco, Madrid, 28049, Spain; 6 Unidad Asociada CNB-Instituto Madrileño de Estudios Avanzados en Nanociencia (IMDEA Nanociencia),Cantoblanco, Madrid, 28049, Spain

## Abstract

We have developed a new data collection method and processing framework in full field cryo soft X-ray tomography to computationally extend the depth of field (DOF) of a Fresnel zone plate lens. Structural features of 3D-reconstructed eukaryotic cells that are affected by DOF artifacts in standard reconstruction are now recovered. This approach, based on focal series projections, is easily applicable with closed expressions to select specific data acquisition parameters.

Cryo soft X-ray tomography (cryo-SXT) is one of the most recent structural biology techniques for quantitative three-dimensional (3D) analysis of whole cells. When preserved in cryo-conditions, whole cells can be imaged by Fresnel zone plate (FZP) lenses with spatial resolution in the few tens of nanometers^1^. Information from these images can then be combined by tomographic techniques to generate 3D maps of the specimen. X-ray photon-energies in the so-called *water window* (between 284 and 543 eV)^2^ are especially useful, since the difference between the linear absorption coefficients (LACs) for carbon (protein) and oxygen (water) allows to obtain high contrast images of cryopreserved whole cells without use of staining agents.

The image formation process in SXT, complex and still under study^3^, combines specific element absorption with marked changes along the optical axis over the whole specimen thickness. In certain conditions, for a region around the specimen and under an incoherent illumination scheme, a detailed formulation was proposed that led to a closed form expression of how images are projected from the 3D density distribution of the specimen^4^. This expression is termed the soft X-ray transform, and a general inversion formula has not been found^5^.

Interest in cryo-SXT has recently increased^6^,^7^,^8^,^9^,^10^,^11^,^12^,^13^,^14^,^15^,^16^. However, images are currently being treated as regular projections with no defocus effects, assuming that specimen thickness is smaller than the optical system depth of field (DOF). However, in the majority of experimental cases, samples are thick, that is, their thickness is larger than the DOF. Therefore, in the case of thick specimens the reconstructed LACs of organelles placed in a region out of the DOF are affected both by a decrease in the contrast and by blurring along z direction in the reconstructed volume, even in the absence of missing wedge^4^.

Here, we present an in-depth mathematical analysis of the soft X-ray transform, leading to its inversion in practical experimental cases. This image processing framework allows a clear understanding of the experimental parameters that affect the quality of the SXT inverse, allowing optimization of data collection. Guided by this analysis, procedures at the Mistral soft X-ray microscope (ALBA Synchrotron Light Source)^17^,^18^ have been implemented to automatically acquire data sets that allow for a more accurate study of thick specimens than standard approaches.

The procedure of XTEND is presented in detail in the Methods section. In brief, several tilt series at different defoci are collected with no radiation damage visible at our spatial resolution. As a general rule, the dose required for a XTEND tilt series is the regular dose used in standard single tomography divided by the number of focal tilt series. Then, the defoci series are averaged and deconvolved using a specific blurring function that derives from the here reported mathematical developments (Eq. 6). The formulas we provide relate the focal step and number of focal series to in-focus z-range and optical system DOF.

## Results

Here we apply our quantitative approach, XTEND, to reconstruct simulated and experimental biological system tomograms in which the specimens thickness are larger than the DOF. Indeed, we present results from samples whose thickness are beyond twice the DOF.

### Simulated results

**Figure 1:**
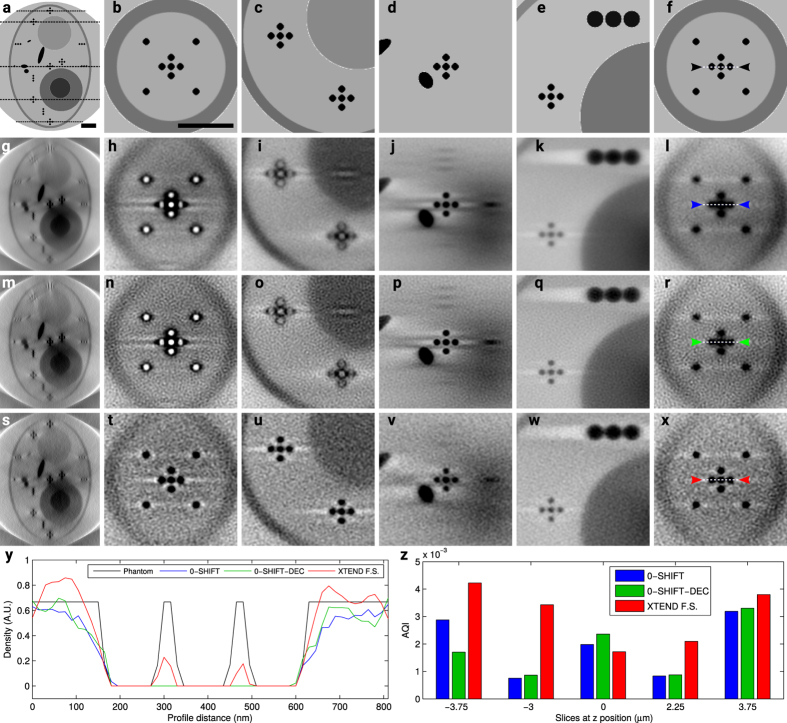
Comparison of the different collection methods on a simulated 9 μm thick pseudo candida albicans cell tomography imaged by a 40 nm FZP in a totally incoherent situation (48.8 nm resolution, 2.63 μm DOF): phantom (first row), 0-shift (second), 0-shift-dec (third) and XTEND (fourth) reconstructions; (**a**,**g**,**m**,**s**) x–z planes where x–y slices at z-positions −3.75 μm (**b**,**h**,**n**,**t**), −3 μm (**c**,**i**,**o**,**u**), 0 μm (**d**,**j**,**p**,**v**), 2.25 μm (**e**,**k**,**q**,**w**) and 3.75 μm (**f**,**l**,**r**,**x**) are marked. Scale bars = 1 μm. (**y**) Density profiles along the paths pointed between color markers (0-shift, 0-shift-dec and XTEND in blue, green and red, respectively) in slices (**l**,**r**,**x**) compared to reference profile (**f**) (black markers). (**z**) AQI calculated for slice triplets (**h**,**n**,**t**), (**i**,**o**,**u**), (**j**,**p**,**v**), (**k**,**q**,**w**) and (**l**,**r**,**x**).

To test our framework, we computed simulations on a phantom equivalent to a whole eukaryotic pseudo cell, which contains pseudo organelles (equivalent to lipid droplets, vacuoles and nucleus). To obtain simulated density values compatible with experimental expectations, we used previously reported LACs to numerically evaluate the 3D function that defines the phantom (Fig. 1(first row))^6^.

To simulate the required set of soft X-ray projections, we used the algorithm described in Otón *et al*.^4^. The algorithm assumes totally incoherent illumination using the 3D point spread function (PSF) that characterizes the optical system of the microscope. In the case of Mistral microscope, two FZPs are available as objective lens. A lower resolution/larger DOF lens, characterized by an outermost zone width of 40 nm; and a higher resolution/lower DOF lens, whose outermost zone width is 25 nm (named hereafter ZP40 and ZP25, respectively). In this simulation we have used the theoretical 3D PSF of ZP40 for a totally incoherent illumination scheme characterized by DOF ~ 2.63 μm and Rayleigh resolution around 50 nm (25 nm half pitch) at 520 eV energy, considering a pixel size of 15 nm. For a thickness phantom design of 9 μm (Fig. 1(a)) and using equation (15), we determined that, to set in-focus a z-range around 8 μm, at least three tilt series separated by 5.5 μm should be collected. The angular range for each tilt series was [−65°, 65°] in 1° steps. Note that current tomographic acquisition schemes would employ a single tilt series, recorded such that the plane of best focus is at the specimen center (focal shift = 0). For purposes of comparison, we will refer to the tomogram obtained using standard reconstruction algorithms as **0-shift** and when projections have been blurring corrected by deconvolution as **0-shift-dec**^19^. Similarly, we will refer as **XTEND** tomogram to the one derived from the combination of the tilt series at different defoci, following the workflow described in this work (see Fig. 7). Gaussian noise was added to the simulated projections for a signal-to-noise ratio (SNR) ≈ 33, to mimic SNR realistic values at 1 s exposition. Note that in the 0-shift cases the simulated exposition time was 3 s to match the SNR for the XTEND case.

We compared distinct slices at the same z positions from the phantom (Fig. 1a–f) with reconstructed slices from the 0-shift (Fig. 1g–l), 0-shift-dec (Fig. 1m–r) and XTEND (Fig. 1s–x) tomograms. Details were much enhanced in the XTEND case, in particular far from the tilt axis, with correction of the phase reversal effect in the quadruplet (b, h, n, t). For quantitative assessment of the XTEND improvement, we analyzed the results in two ways. In Fig. 1y we plotted the intensity profiles along the paths pointed by color marks in slices at 3.75 μm in *z* from the tilt axis position (f, l, r, x), which show that XTEND clearly enhanced the contrast compared to standard collection and reconstruction. We also applied a blind image quality assessment (AQI)^20^ to evaluate absolute image quality without a reference among corresponding planes from the 0-shift, 0-shift-dec and XTEND tomograms (Fig. 1z). The recovered information measured this way was always greater in the XTEND reconstruction than in the 0-shift case, except for the central slice, where 0-shift-dec showed the best result. It means that the application of XTEND did not enhance the reconstruction in the central region as it was already in focus and the lowpass filter deconvolution did not completely recover the blurred information. Nevertheless, the global performance of XTEND at distant regions compensated for the small quality loss in the central region. Note that at −3.75 μm slice AQI shows that 0-shift is better reconstructed than 0-shift-dec, remarking that standard deconvolution applied in 0-shift-dec cannot correct for the contrast inversion at those distances.

The simulation results shown in Fig. 1 validate the use of XTEND in an incoherent optical system. However, the experimental characterization of both FZPs used at the Mistral microscope show a response that differs from ideal^19^. Although theoretical designs resulted in a totally incoherent and partially coherent regimes for ZP40 and ZP25, respectively, experimental characterization of the optical system for each scheme showed that the effective illumination pattern lead to a lower effective numerical aperture of the condenser (and, therefore, a greater degree of coherence), resulting in a Rayleigh resolution of 61.9 and 51.8 nm and DOF of 3.3 and 1.6 μm for ZP40 and ZP25, respectively^19^. Thus, for a more realistic validation we also computed tomogram simulations considering 3D PSF distributions numerically calculated to match both DOF and resolution for each experimental ZP. On one hand, for the experimental ZP40 case, we have used the same phantom as for theoretical ZP40 (Fig. 1), obtaining the same z-step and number of focal series projections. Results are shown in Supplementary Fig. 1, which follow a similar behaviour, although not identical, to the theoretical ZP40 ones. We note the contrast inversion effect in slices at −3 μm was less intense for experimental ZP40 due to the enlarged DOF. In addition, the AQI shows slightly better quality data with XTEND than with 0-shift in the central region compared to the theoretical ZP40 case. On the other hand, for the experimental ZP25 case, we scaled our phantom, setting a pixel size of 10 nm and keeping the selected slices, to match a 5.5 μm thick cell. In this case, we obtained, for 3 focal series, a 3.12 μm z-step should be used. As in both ZP40 cases (theoretical and experimental), results showed that XTEND performance recovered more information in regions far from the tilt axis, even more noticeable as the ratio phantom thickness/DOF was even greater than the ones in ZP40 cases (Supplementary Fig. 2).

## Experimental Results

We proceeded analyzing XTEND performance in two experimental datasets acquired at the Mistral microscope^18^. The first reconstruction was obtained of a *Scenedesmus* cell using ZP40 (*Scenedesmus* is a phototrophic microorganism isolated from Ebro delta (Spain) microbial mats in 2009), while the second case is a reconstruction of a HT-29 cell using ZP25. Note that the total photon dose used for experimental XTEND tilt series was below the damage limit at our achievable resolution. However, it was not possible to acquire an extra focal series at 0-shift to compare it with XTEND at same SNR levels without exceeding the damage limit as in the simulated tomograms.

Previous analysis of a *Sc.* cell shaped a z-range of interest ~6 μm, while the DOF measured for ZP40 was 3.3 μm^19^. We thus determined that, for a total of 3 focal series, a z-step ~4 μm should be used for correct application of XTEND (equation (15)). Collection geometry was single tilt axis in the range [−70°, 68°] in 1° steps, with 1 s exposure time and 13 nm pixel size. Note that focal series projections were obtained by moving the FZP along the optical axis at each tilt angle and, although showed small shift misalignment around 1–2 pixels in *x* and *y* axes due to the ZP motion, it was corrected by comparison to the reference 0-shift tilt series using EFTEM-TomoJ^21^. The average of the focal series projection at each tilt angle was aligned by IMOD^22^. The in-focus z-range value of 6 μm and a SNR estimation for the 0-shift and XTEND projections, near 20 and 100, respectively, were used as input for deconvolution.

**Figure 2 f2:**
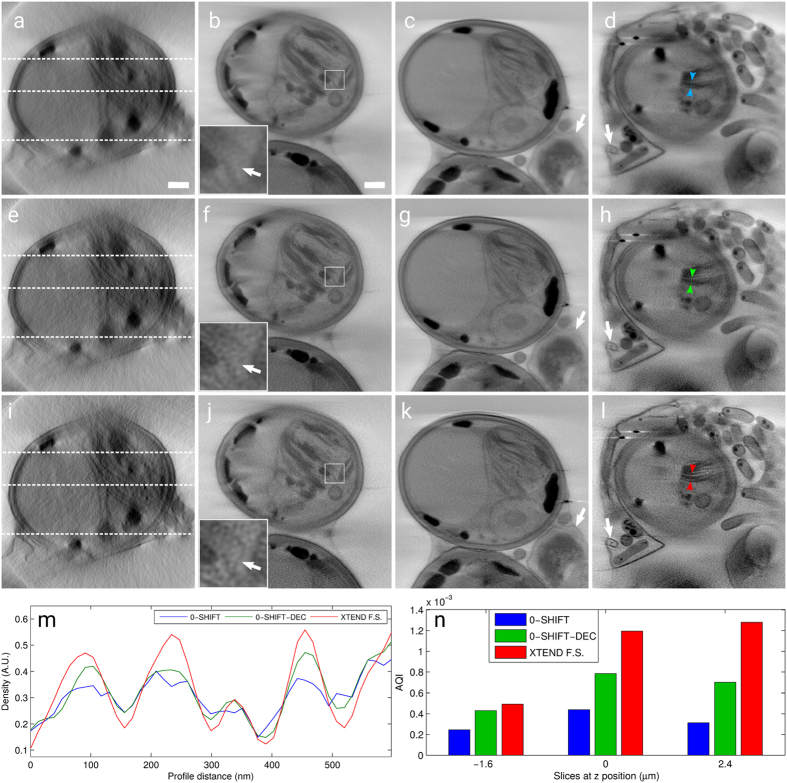
Comparison of the different collection methods on a *Scenedemus* cell experimental tomography imaged by a 40 nm FZP at Mistral microscope (61.9 nm resolution, 3.3 μm DOF): 0-shift (first row), 0-shift-dec (second) and XTEND (third) reconstructions; (**a**,**e**,**i**) x–z planes where x–y slices at positions −1.6 μm (**b**,**f**,**j**), 0 μm (**c**,**g**,**k**) and 2.4 μm (**d**,**h**,**l**) are marked. Scale bars = 1 μm. (**m**) Density profiles along the paths pointed between color markers (0-shift, 0-shift-dec and XTEND in blue, green and red, respectively) in slices (**d**,**h**,**l**). (**n**) AQI calculated for slice triplets (**b**,**f**,**j**), (**c**,**g**,**k**) and (**d**,**h**,**l**).

Figure 2 compares slices of the three reconstruction procedures, 0-shift, 0-shift-dec and XTEND, with objective quality measures as for the simulations. Experimental data sections of the reconstructed XTEND volume already show a significant improvement, especially for internal features of cellular organelles, as those ones pointed by white arrows (Fig. 2j–l). Density profiles extracted from slices at 2.4 μm are plotted in Fig. 2m. They clearly show the highest contrast enhancement by XTEND compared to standard 0-shift and 0-shift-dec. The AQI measurement shows a clear quality increase for all planes, considering the comparison conditions we can achieve experimentally regarding dose limitation between 0-shift and XTEND cases (Fig. 2n). In this case, 0-shift-dec did not show the greatest quality index at central slice, probably due to a slight misplacement of the central tilt axis close to −0.8 μm instead of 0 μm.

**Figure 3 f3:**
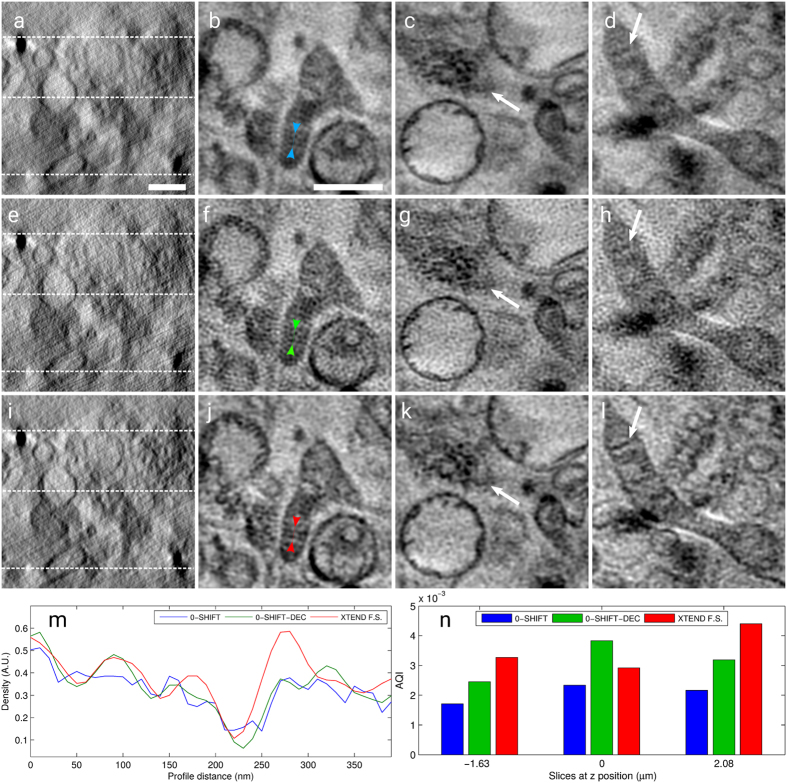
Comparison of the different collection methods on a HT-29 cell experimental tomography imaged by a 25 nm FZP at Mistral microscope (51.8 nm resolution, 1.6 μm DOF): 0-shift (first row), 0-shift-dec (second) and XTEND (third) reconstructions; (**a**,**e**,**i**) x–z planes where x–y slices at positions −1.63 μm (**b**,**f**,**j**), 0 μm (**c**,**g**,**k**) and 2.08 μm (**d**,**h**,**l**) are marked. Scale bars = 1 μm. (**m**) Density profiles along the paths pointed between color markers (0-shift, 0-shift-dec and XTEND in blue, green and red, respectively) in slices (**d**,**h**,**l**). (**n**) AQI calculated for slice triplets (**b**,**f**,**j**), (**c**,**g**,**k**) and (**d**,**h**,**l**).

The second experimental tilt series, shown in Fig. 3, is a sample of HT-29 cells. It was acquired with the high resolution zone plate ZP25. In this case, the estimated z-range of interest was ~5 μm, equivalent to its cytoplasm and combined with the experimental DOF of 1.6 μm, resulted in 3 focal series and z-step of ~3.3 μm. The tilt geometry in this experiment was in the range [−65°, 65°] in 1° steps, with variation of exposure time between 2 and 3 s and 10 nm pixel size. A clear improvement in the mitochondria cristae is noticeable in the XTEND reconstruction. Although the use of ZP25 is characterized by a higher degree of coherence than the one for ZP40, the results in Fig. 3 demonstrate a similar behaviour when XTEND is applied, probably because both cases are partially coherent.

## Discussion

We have described XTEND, a data collection and image processing workflow for cryo-SXT applied to biological systems that reduces the shortcomings associated with the limited depth of field of the optical system. It combines image projections at different focal distances and applies a deconvolution algorithm to correct for the lowpass filter inherent to the method.

The practical parameters for application of the technique are compiled in a simple formula. It minimizes the number of focal series projections needed to fully compensate for the limited DOF. Therefore, XTEND can be applied to any type of biological sample since the total photon dose is not increased.

Results showed a clear enhancement of blurred regions far from the tilt axis when sample thickness was around twice the DOF for lower resolution ZP40 and 3 times the DOF for ZP25.

XTEND does not introduce any reconstruction enhancement in the central region of the sample tomogram, always in focus, compared to the 0-shift-dec, as it is affected by the lowpass filter introduced by XTEND. Future works may focus on correcting this quality decrease in the central part, by combining information from both 0-shift-dec and XTEND volumes according to a local quality parameter. Even more, this effect may be prevented by specifically applying XTEND only at those tilt angles whose sample cross section is larger than DOF.

Future applications in the field of cryo-SXT point to the use of FZPs with higher resolution and shorter DOF, making the application of XTEND a necessity.

## Methods

The inversion of the soft X-ray transform has, in general, resisted solution^23^. Simple analysis of the nature of soft X-ray imaging indicates several practical issues that must be considered for stable inversion. One crucial problem is that each plane of the specimen is affected by a different blurring function; when combined with absorption and the ever-present experimental noise, this generates a complicated image. For specimens thicker than the DOF in practical cases, some sample planes will be so blurred that faithful information recovery can be nearly impossible. Combining information from images obtained at different defoci would thus appear evident, whereby features that are blurred at a certain focus are sharper at others.

In the literature we find different proposals to correct for the DOF effects in the projection image: wavelet-based algorithms to obtain composite 2D images through a focal series approach, in transmission X-ray microscopy^24^ and in the area of bright field microscopy^25^. A recently proposed method in X-ray microscopy reconstructs the 3D information using a multi-focus approach that requires specific algorithms as well as precise measurement of the relative position of the best focusing plane over specimen thickness^26^. However, in all these cases, the algorithms only qualitatively enhance information contrast in projections and 3D volumes without quantitatively recovering the linear absorption coefficients.

Here we present a mathematical derivation that leads to a practical approach to the inverse of SXT data. In Fourier space, we elaborate the representation of a specific image obtained in the soft X-ray microscope at a given focus from a known projection direction; introducing the mathematical notation that will be used hereafter.

### Fourier space representation of an image projection

To analyze in Fourier space which sample information is contained in a given image, let us consider the image formation model for totally incoherent illumination and z-variant point spread function (PSF)^4^.


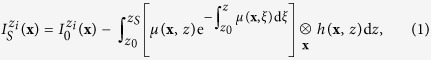


where **x** = (*x,y*), 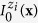 and 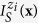 are the projections acquired without and with the sample, respectively*; μ*(**x**,*z*), with 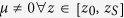, is the volume that describes the 3D distribution of the sample absorption coefficients, *h*(**x**,*z*) is the corresponding PSF at each *z* plane and 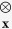 denotes the convolution operation in (**x**). This equation (1) leads to the following reduced expression:


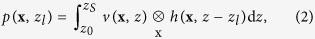


where 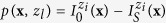 is the reduced projection for a given *z*_*l*_ focal shift between the sample and the best focusing plane, and 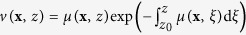 is the reduced volume.

If we apply the Fourier transform to equation (2) we obtain:


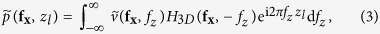


where **f**_x_ = (*f*_*x*_,*f*_*y*_), 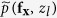 is the 2D Fourier transform of projection *p*(**x**,*z*_*l*_) (in **x** plane), 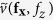 is the 3D Fourier transform of reduced volume *v*(**x**,z) and *H*_3*D*_(**f**_**x**_,*f*_*z*_) is the 3D Fourier transform of the PSF *h*(**x**,*z*), also known as 3D-OTF^27^.

We observe that a soft X-ray image projection, which is expressed as an integration over the *z* axis in real space of all z-slices convolved by their corresponding PSF (equation (2)), is also an integration over the *f*_*z*_ axis in Fourier space (equation (3)). This last equation shows that the Fourier coefficient at transverse frequency **f**_**x**_ of a projection is the addition of all coefficients of the specimen along axial *f*_*z*_ at that same transverse frequency. As the 3D-PSF is a band-limited function, this integration is limited by the cut-off frequency *f*_*zc*_. This cut-off frequency over the *f*_*z*_ axis is related to the transverse in-plane cut-off frequency *f*_*xc*_ of a totally incoherent system such as 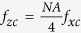
28, where *NA* is the numerical aperture of the objective lens. If we replace this with 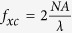 for totally incoherent illumination with wavelength λ, we evaluate the limit as


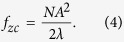


**Figure 4 f4:**
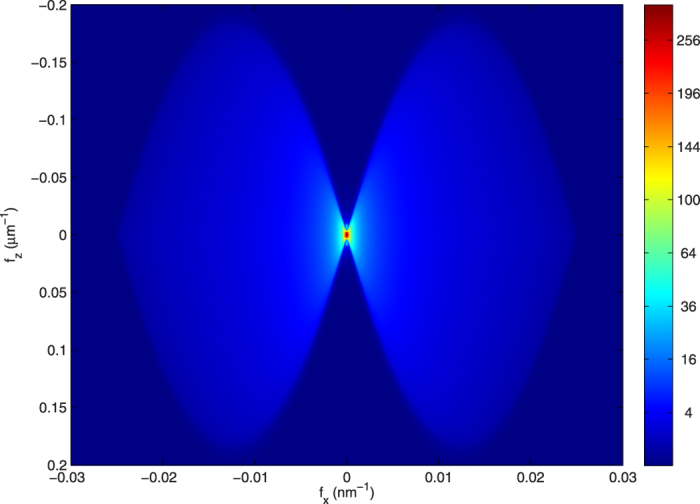
Section of an ideal 3D-OTF in the plane *f*_*x*_ − *f*_*z*_. Simulation was calculated for an ideal lens under totally incoherent illumination, with same characteristics as a zone plate with 40 nm of outermost zone width, 937 zones and 520 eV illumination energy. Both lobes of the figure mark the specimen Fourier coefficients over *f*_*z*_ that contribute to the projection, which is calculated by integration over *f*_*z*_.

In Fig. 4 we show a section of a theoretical 3D-OTF *H*_3*D*_ along *f*_*z*_ and **f**_**x**_, calculated for an ideal ZP with 40 nm of outermost zone width under incoherent illumination at 520 eV with a limit *f*_*zc*_ = 0.186 μm^−1^, where both lobes of the figure mark the Fourier coefficients of the specimen that contribute to the projection. In the case in which the specimen is totally in focus, it is accepted that 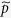 follows the *central slice theorem*^29^, which means that the relative size of *H*_3*D*_ along *f*_*z*_ is much smaller than the size of 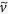. For specimens larger than the DOF, the *f*_*z*_ size ratio is inverted and coefficients far from the central slice are collected, which are those responsible for blurring in the projection image. The projection of an object whose thickness *T*_*zDOF*_ is around the corresponding DOF 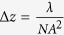
30, and therefore, completely in-focus, is thus obtained from integration over *f*_*z*_ in the band [−*f*_*zc*_,*f*_*zc*_] given by


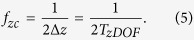


### Collecting information for a stable SXT inverse: the continuous case

In the theoretical case that an extremely large number of projections could be acquired, without damaging the sample, at infinitesimally small z-steps dz, then equation (3) leads to


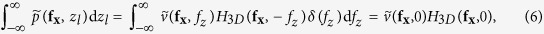


which shows that the addition of projections at different *z*_*l*_ values equals the central slice of 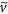 multiplied by the central slice of the 3D-OTF *H*_3*D*_. The ideal continuous case, therefore, produces the same result as a projection of a totally in-focus sample convolved by a highly smooth low-pass filter, which can then be deblurred. After applying deconvolution, the use of standard reconstruction algorithms will lead to a corrected reconstruction.

### Collecting information for a stable SXT inverse: the discrete and limited case

As the previous case requires collection of an infinite number of images at all defoci, which is experimentally impossible, limitations on acquisition of the set of z projections must be considered. Here we analyze a practical case, for which only a few images at different defoci can be obtained. If we add a finite number of *M* focal series projections with a z-step *L* along the z-positions *z*_*min*_ and *z*_*max*_ 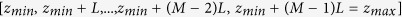 symmetrically distributed around *z* = 0, we obtain


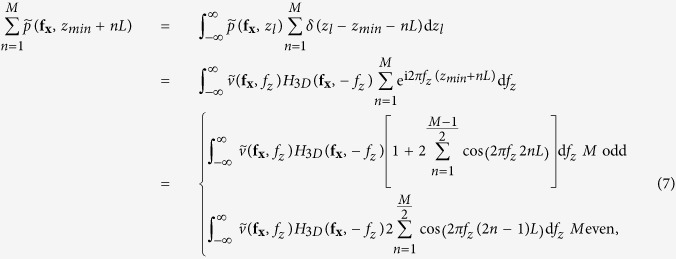


that, in the limit case when *M* ≫ 1, becomes


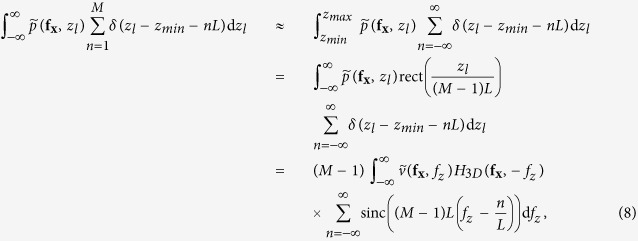


where 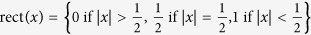 is the rectangle function and 
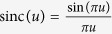
 is the Fourier transform of rect(*x*). Equation (8) shows that the Fourier coefficients of the averaged projection are the addition of Fourier coefficients of the specimen around fixed positions 
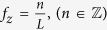
, whose envelope is determined by the sinc function. Hence, we can choose an adequate z-step to place secondary orders of n beyond the range of the 3D-OTF:


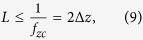


and, then, only Fourier coefficients around *f*_*z*_ = 0 (n = 0) will contribute. Setting *L* < 2 Δ*z* clearly does not improve effective reconstruction. However, from equation (5), we can calculate for a specimen of any thickness *T*_*z*_ the Fourier range 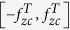, whose integration is equivalent to the central slice, by


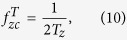


and then, as seen on equation (8), it is possible to adjust the range over *f*_*z*_ by the sinc function envelope. The position of the first sinc zero around *f*_*z*_ = 0 (n = 0) is given by 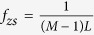 and therefore, if we set


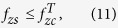


we obtain an averaged focal series projection whose *f*_*z*_ range corresponds to a totally in-focus specimen. Equation (8) is valid for *M* ≫ 1, but *M* is intended to be as small as possible, in which case the shape of the envelope is not close to a sinc function but to the cosine functions addition shown in Eq. 7. It can be proved that, from the smallest value of *M* = 2 (that leads to a single cosine envelope along *f*_*z*_) to *M* ≫ 1, the position of the first zero of the envelope as function of *M* is given by


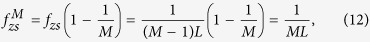


and substituting onto equation (11) along with equation (10), we obtain the minimum value for the number of focal series projections needed to fulfill equation (6)


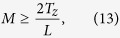


and the total scanned length along the optical axis *z* to be covered by the focal series is


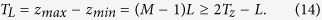


This development leads to an apparent counterintuitive result: as *T*_*z*_ increases compared to *L*, to extend the DOF fully to the sample thickness, we must scan a greater distance. This can be understood qualitatively as the same range of defocus patterns is needed for every slice of the specimen to allow equal correction for the *H*_3*D*_ central slice (equation (6)). Since the DOF distance is a criteria threshold, however, we can consider a correction factor *α* that reduces the scanned total length by guaranteeing that at least a *T*_*z*_*α* range, with *α* ≤ 1, is completely in the depth of field. We can then calculate the allowed minimum number of focal series projections, *M*, by selecting the maximum value for z-step *L* = 2Δ*z*,(equation (9)). The lowest value for *M* therefore becomes


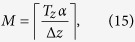


where the *ceiling* function 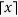 denotes the nearest integer ≥*x*. To distance secondary orders of sinc function in equation (8) we select this minimum *M* value to obtain its z-step:


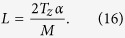


**Figure 5 f5:**
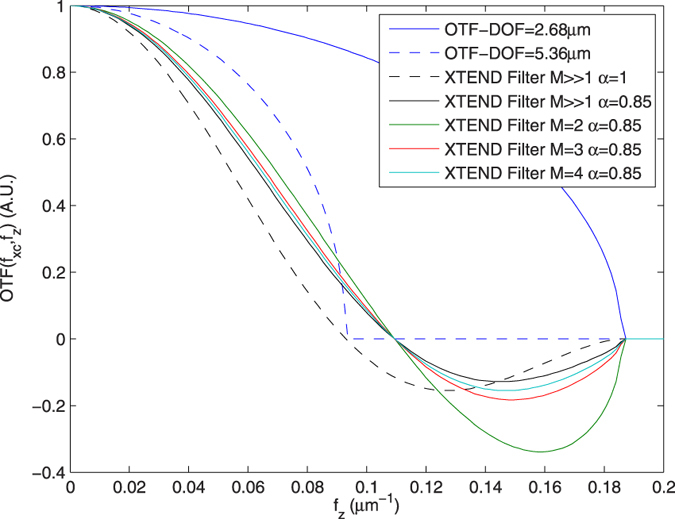
Z-axis contributing Fourier coefficients in a projection of a sample whose thickness is twice the DOF of the optical system. Solid blue line denotes the 3D-OTF profile over the *f*_*z*_ axis at 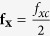 of the optical system (see Fig. 4). The profile of the sample to be considered totally in-focus is defined by the 3D-OTF profile of a lens with twice the DOF of the optical system (dashed blue line). The resulting ideal sinc envelope profile for *M* ≫ 1 focal series projections is depicted by a dashed black line. Envelope profiles using correction factor *α* = 0.85 for *M* = 2,3,4 and *M* ≫ 1 are plotted in solid green, red, cyan and black lines, respectively. The use of only two focal series projections introduces an inversion of contrast at high frequencies. When *M* = 3,4, however, the resulting envelopes differ slightly from that of the ideal *M* ≫ 1.

We observe that the total scanned length is 
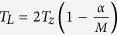
 that, in the limit when *M* ≫ 1, is twice *T*_*z*_. Moreover, the effect of considering a correction factor *α* to reduce the number of focal series projections forces enlargement of the total scanned length. For instance, Fig. 5 shows a simulation for an observed object whose thickness is twice the DOF of the objective lens. For the objective lens, we plot the profile of *H*_3*D*_ from Fig. 4 at **f**_**x**_ = 0.0125 nm^−1^ along *f*_*z*_ as a solid blue line. This is compared to the same profile of a lens with twice the DOF, which identifies the range for the object as if it were in-focus (dashed blue line). Ideally, the use of a large number of focal series projections would lead to the envelope profile of the sinc function, depicted as a dashed black line. In a more realistic scheme, for a correction factor *α* = 0.85, we plot the envelope profiles for a number of focal series projections of *M* = 2,3,4 and *M* ≫ 1 (solid green, red, cyan and black lines), with a total scanned length of 1.15, 1.43, 1.58 and 2 times the thickness of the specimen, respectively; when only two focal series projections are used, a contrast inversion appears at high frequencies; when three or more focal series projections are used the solution is not far from the limit given by *M* ≫ 1.

### Data collection procedure: practical considerations

From a theoretical point of view, the proposed technique produces better results as the number of focal series projections increases. In practice, however, radiation damage of the sample must be avoided which, in general, requires a dose fractionation such that the total dose over the specimen is divided over the total set of tilt series at different defoci.

For accurate information recovering, the microscope transfer function must be measured for precise knowledge of system magnitudes; this also provides a correct value of z-step *L* to be used in the focal series acquisition, as well as the entire 3D-OTF to deconvolve the averaged projections. The optical system of Mistral microscope is composed of a single bounce glass capillary condenser, characterized by a length of 100 mm, with inner entrance and exit diameters of 1.82 and 0.58 mm, respectively, that works as a single reflection achromatic lens with a focal length of 10.05 mm. The two available FZP objective lenses, ZP40 and ZP25, are characterized by outermost zone widths of 40 and 25 nm, 937 and 1,500 zones, respectively, that give, at 520 eV photon energy, 2.52 and 1.57 mm theoretical focal lengths and *NA*_*cond*_/*NA*_*obj*_ ratios 1 and 0.63, respectively. However, these lenses are not the only optical elements in the microscope and manufacturing processes are rather complicated leading the optical system impulse response to diverge from the ideal one. In this way, we measured the apparent transfer function of the typical optical schemes of the Mistral soft X-ray transmission microscope using a Siemens star test pattern^19^,^31^.

Once the DOF is known, z-step must be calculated before the acquisition of the focal series projections. Considering ZP40 DOF 3.3 μm and a sample estimation ~6 μm, equation (15) lead to a value of *M* = 3 focal series projections, acquired at *z*_*l*_ = −4,0 and 4 μm. In the case of ZP25, 1.6 μm DOF, a sample estimation ~5 μm and *α* = 0.9 resulted in *M* = 3 and z-step of 3.3 μm.

**Figure 6 f6:**
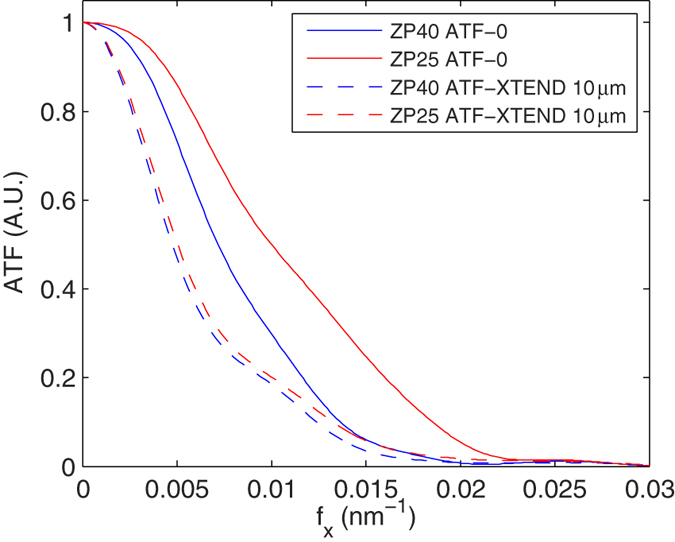
ATF-0 profiles measured for ZP40 and ZP25 compared to the synthetic ATF-XTEND profiles calculated from the experimental 3D-PSF of the FZPs for a 10 μm z-range.

After combination of the focal series by averaging, the multiplexed projections were corrected for the low-pass filter given by the central plane of *H*_3*D*_ at *f*_*z*_ = 0 (equation (6)), termed ATF-XTEND. This ATF-XTEND profile is the addition of all ATF profiles over z. The analytical calculation of ATF-XTEND from the 3D Fourier transform of the 3D-PSF expression of an ideal lens is not possible due to a singularity in the origin (**f**_**x**_ = 0,*f*_*z*_ = 0). In certain approximations, however, we find an analytical expression that depends on sample thickness^28^. Therefore, the 3D-PSF characterization for the z-range beyond the sample thickness, which includes both sample and focal series shifts, is required for a proper deconvolution. Even to obtain the ATF-XTEND profile from experimental 3D-PSF data, we have to estimate the in-focus z-range first from, for example, *a priori* knowledge or by reconstructing a standard tomogram (*z*_*l*_ = 0). Figure 6 Shows that the attenuation introduced by ATF-XTEND profile compared to ATF-0 for the ZP40 case is lower than the one for ZP25, due to the difference in the DOF. However, we observe in both cases a clear attenuation in middle frequencies, around 0.01 nm^−1^. Finally, ATF-XTEND profiles are use to deconvolve the averaged projections by applying a Wiener filter.

### A quantitative soft X-ray tomography map: a practical workflow

**Figure 7:**
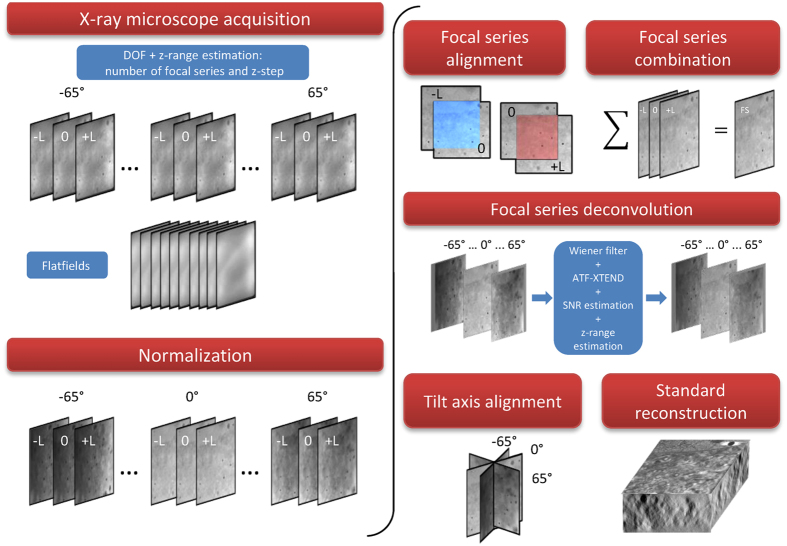
XTEND Workflow: Microscope DOF and in-focus z-range estimation are used to calculate number of focal series projections and z-step. For 3 focal series projections, images are acquired at z-positions −L, 0 and +L. Image projections are normalized using flatfield images. Focal series projections −L and +L are aligned to reference 0-shift at each tilt angle and combined by averaging. Averaged projections stack is deconvolved by synthetic ATF-XTEND considering z-range and the SNR from projections and flatfields. Once deconvolved, the XTEND projections are then aligned to the common tilt axis and reconstructed using standard algorithms.

The entire process using the Mistral microscope, from data acquisition to final focal series reconstruction, is depicted in Fig. 7. After selecting the region of interest in the sample grid, a coarse estimation of the z-range results in the number of focal series and z-step parameters. Then, acquisition of the focal series projections, automated by macro functions, is implemented. At each tilt angle, the different z-projections are recorded sequentially by shifting the FZP lens. A set of ten flatfield projections is acquired to normalize data projections and to evaluate the SNR value for the Wiener filter algorithm. For normalization by flatfield correction, also considering exposure time and beam intensity, we used XMIPP3.1^32^.

Although the z-focus process is implemented to minimize misalignment among z projections, a small shift error in *x* and *y* axes remains of around 1–2 pixels. To correct for this misalignment, secondary focal series stacks were compared to the reference stack and realigned (EFTEM-TomoJ^21^). Note that the variation of the optical system magnification among the distinct focal planes, <1 pixel, is considered negligible. The aligned focal series projections at each tilt angle were combined by averaging. These averaged projections were deconvolved using as kernel the transfer function ATF-XTEND, calculated from the experimental 3D-ATF profiles of the FZP optimized for the in-focus z-range. Then, the averaged projections were aligned to the common tilt axis, facilitated by sharpen structures using landmarks, in IMOD^22^ (1 pixel error). To adapt X-ray projections for use of standard reconstruction algorithms^5^, the Napierian logarithm was applied to projections to, finally, reconstruct using the iterative algorithm SIRT (30 iterations) implemented in TOMO3D^33^.

## Additional Information

**How to cite this article:** Otón, J. *et al*. XTEND: Extending the depth of field in cryo soft X-ray tomography. *Sci. Rep.* **7**, 45808; doi: 10.1038/srep45808 (2017).

**Publisher's note:** Springer Nature remains neutral with regard to jurisdictional claims in published maps and institutional affiliations.

## Supplementary Material

Supplementary Figures
