# Supplementary material for: XTEND: Extending the depth of field in cryo soft X-ray tomography
Source: Sci Rep. 2017 Apr 4;7:45808. doi: 10.1038/srep45808 (PMC5379191; doi:10.1038/srep45808)
Supplement: Supplementary Figures [file srep45808-s1.pdf]

Supplementary Information

XTEND: Extending the depth of field in cryo soft X-ray tomography

Joaquín Otón[1,*], Eva Pereiro[2], José J. Conesa[1], Francisco J. Chichón[1], Daniel Luque[3], Javier M. Rodríguez[3], Ana J. Pérez-Berná[2], Carlos Oscar S. Sorzano[1], Joanna Klukowska[4], Gabor T Herman[4], Javier Vargas[1], Roberto Marabini[5], José L. Carrascosa[1,6], and José M. Carazo[1]

[1] Centro Nacional de Biotecnologìa (CNB-CSIC), Cantoblanco, 28049, Madrid, Spain
[2] ALBA Synchrotron Light Source, Cerdanyola del Vallès, 08290, Barcelona, Spain
[3] Centro Nacional de Microbiología, ISCIII, Majadahonda, 28220, Madrid, Spain
[4] Department of Computer Science, The Graduate Center, City University of New York, NY 10016, USA
[5] Escuela Politecnica Superior, Univ. Autonoma de Madrid, Cantoblanco, 28049, Madrid, Spain
[6] Unidad Asociada CNB-Instituto Madrileño de Estudios Avanzados en Nanociencia (IMDEA Nanociencia), Cantoblanco, 28049 Madrid, Spain
[*] joton@cnb.csic.es

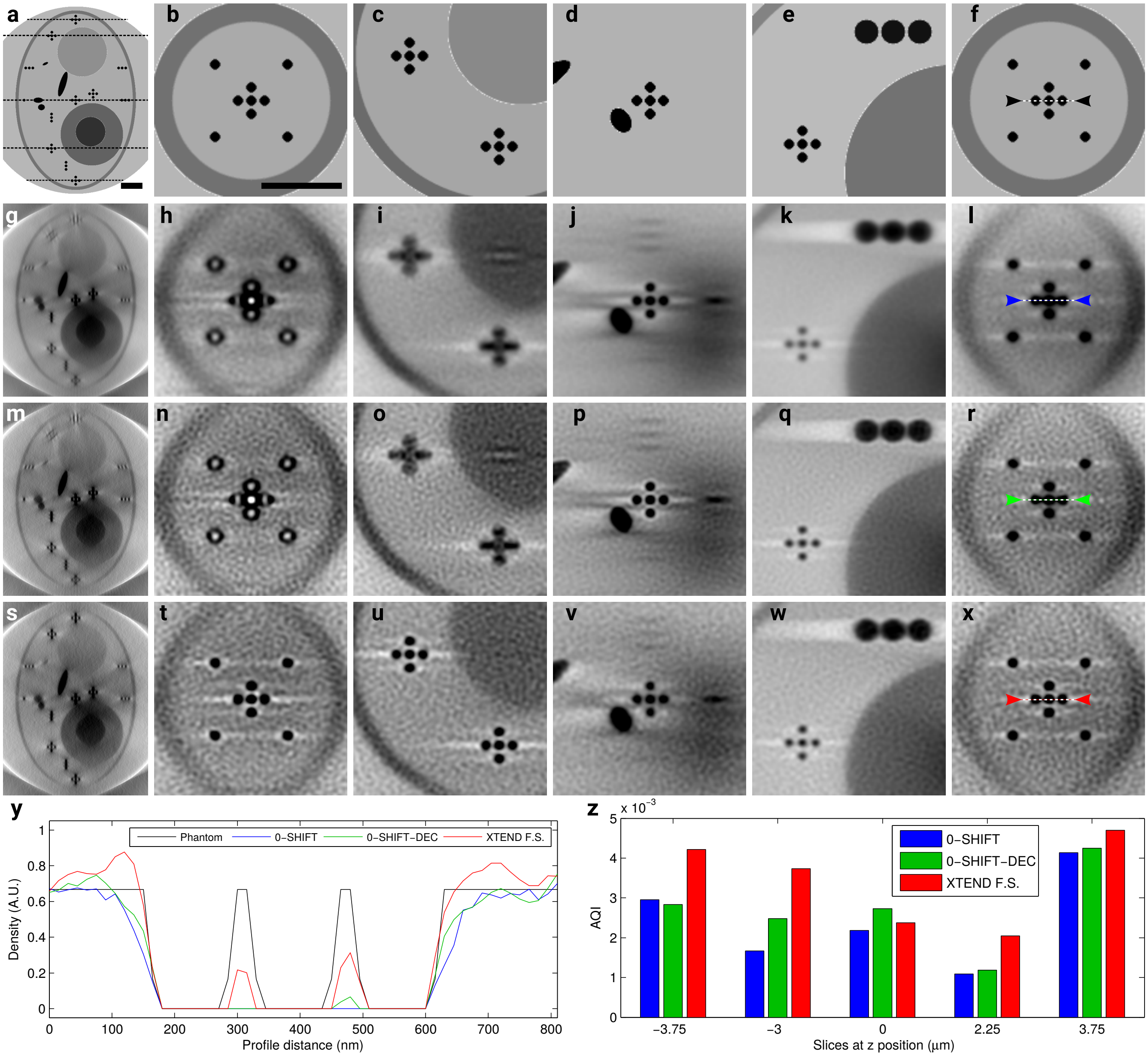

Supplementary Figure S1. Comparison of the different collection methods on a simulated 9 µm thick pseudo candida albicans cell tomography imaged by a 40 nm FZP in Mistral microscope (61.9 nm resolution, 3.3 µm DOF): phantom (first row), 0-shift (second), 0-shift-dec (third) and XTEND (fourth) reconstructions; (a, g, m, s) x-z planes where x-y slices at z-positions -3.75 µm (b, h, n, t), -3 µm (c, i, o, u), 0 µm (d, j, p, v), 2.25 µm (e, k, q, w) and 3.75 µm (f, l, r, x) are marked. Scale bars = 1 µm. (y) Density profiles along the paths pointed between color markers (0-shift, 0-shift-dec and XTEND in blue, green and red, respectively) in slices (l, r, x) compared to reference profile (f) (black markers). (t) AQI calculated for slice triplets (h, n, t), (i, o, u), (j, p, v), (k, q, w) and (l, r, x).

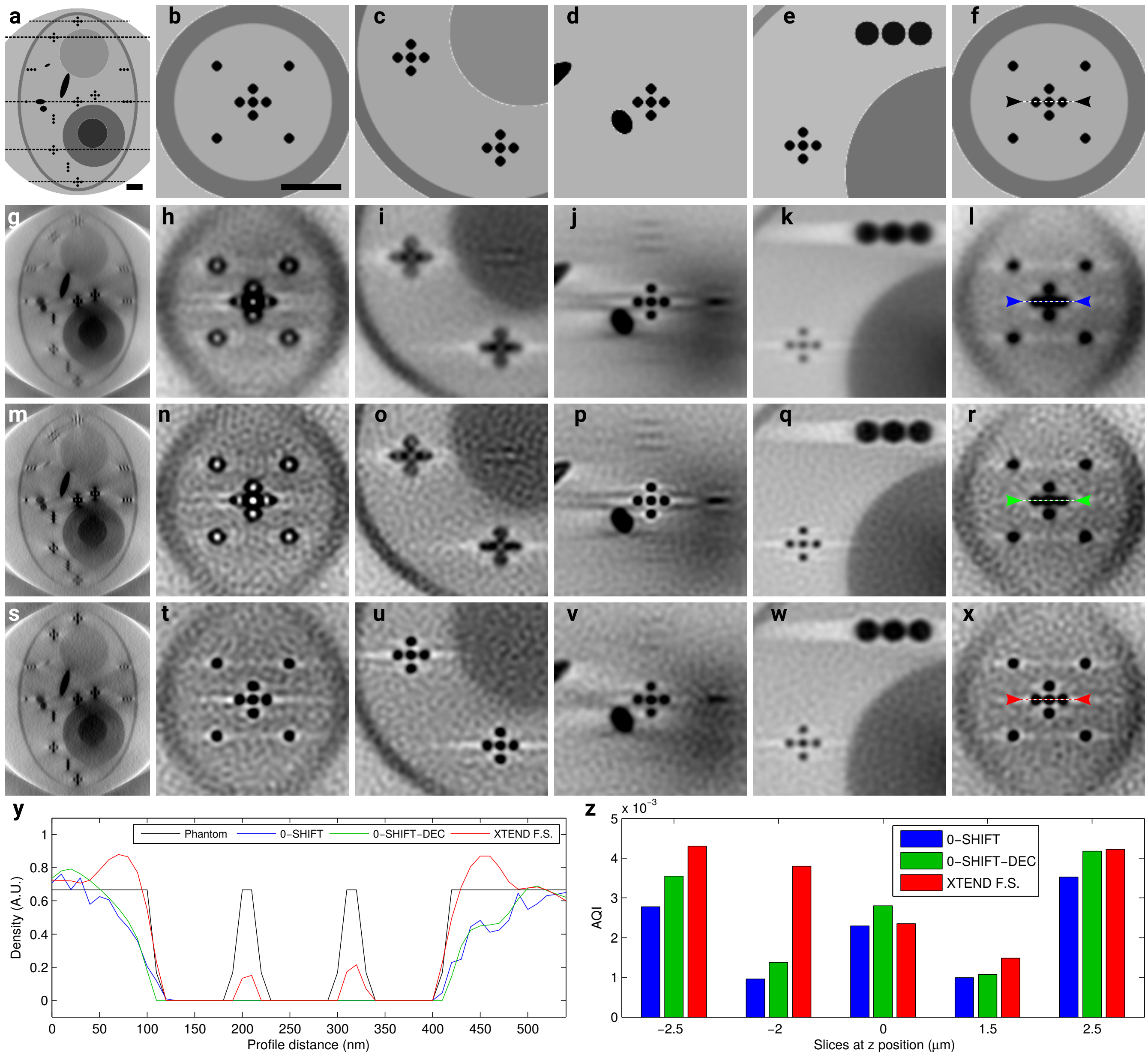

Supplementary Figure S2. Comparison of the different collection methods on a simulated 5.5 µm thick pseudo candida albicans cell tomography imaged by a 25 nm FZP in Mistral microscope (51.8 nm resolution, 1.6 µm DOF): phantom (first row), 0-shift (second), 0-shift-dec (third) and XTEND (fourth) reconstructions; (a, g, m, s) x-z planes where x-y slices at z-positions -2.5 µm (b, h, n, t), -2 µm (c, i, o, u), 0 µm (d, j, p, v), 1.5 µm (e, k, q, w) and 2.5 µm (f, l, r, x) are marked. Scale bars = 500 nm. (y) Density profiles along the paths pointed between color markers (0-shift, 0-shift-dec and XTEND in blue, green and red, respectively) in slices (l, r, x) compared to reference profile (f) (black markers). (t) AQI calculated for slice triplets (h, n, t), (i, o, u), (j, p, v), (k, q, w) and (l, r, x).
